# Degradation of Single Stranded Nucleic Acids by the Chemical Nuclease Activity of the Metal Complex [Cu(phen)(nal)]^+^

**DOI:** 10.1155/S1565363303000025

**Published:** 2003

**Authors:** Norma Ramírez-Ramírez, Guillermo Mendoza-Díaz, Mario Pedraza-Reyes

**Affiliations:** 1 Instituto de Investigación en Biología Experimental. Edificio “L” Facultad de Química Universidad de Guanajuato Apartado Postal 187. Noria Alta S/N Guanajuato 36050 Guanajuato Mexico; 2 Posgrado en Química Inorgánica Facultad de Química Universidad de Guanajuato Apartado Postal 187 Guanajuato 36050 Mexico

## Abstract

The chemical design of metal complexes of the type [Cu(phen)(antib)]^+^ (where antib is a quinolone or a fluoroquinolone) has been carried out in an approach to better understand how the coordination of their components affect the activity of quinolones. The ability of [Cu(phen)(nal)]^+^ to interact with DNA in vivo and its capacity to promote the degradation of plasmid and chromosomal DNA, under reductive conditions has been previously reported. However whether this compound utilizes other intracellular targets to promote bacterial killing was a question that deserved to be answered. In this paper, the studies of the chemical nuclease properties encoded by the metal complex [Cu(phen)(nal)]^+^ were extended by using different types of single chain nucleic acids, i.e, ribosomal and tumor mosaic virus RNAs as well as poly-dA-dT. Our results showed that degradation of the nucleic acids occurred only under reductive conditions. Although MPA and [3-mercaptoethanol were the chemical reducers that best assisted the nuclease reaction, other biological compounds such as citric and succinic acid also were shown to act like reducers in that reaction. All.hough the nuclease activity of [Cu(phen)(nal)]^+^ was comparable to that exhibited by bis copper phenanthroline [Cu(phen)z]^2+^our results showed that none of the individual components of [Cu(phen)(nal)]^+^ was able to promote the degradation of either the RNAs or poly(dA-dT). These results strongly support the hypothesis that the metal complex [Cu(phen)(nal)] uses not only DNA but also RNA as targets to promote bacterial killing.


### Degradation of Single Stranded Nucleic Acids by the Chemical Nuclease Activity of the Metal Complex $[Cu(phen)(nal)]^+$

Norma Ramírez-Ramírez[1], Guillermo Mendoza-Díaz[2], Mario Pedraza-Reyes[1,] ✉

[1]*Instituto de Investigación en Biología Experimental. Edificio "L", Facultad de Química. Universidad de Guanajuato. Apartado Postal 187. Noria Alta S/N. Guanajuato, 36050 Guanajuato, Mexico*

[2]*Posgrado en Química Inorgánica. Facultad de Química. Universidad de Guanajuato. Apartado Postal 187. Guanajuato, Guanajuato, 36050. Mexico*

(Received: June 19, 2002; Accepted: August 20, 2002)

**ABSTRACT**

The chemical design of metal complexes of the type $[Cu(phen)(antib)]^+$ (where antib is a quinolone or a fluoroquinolone) has been carried out in an approach to better understand how the coordination of their components affect the activity of quinolones. The ability of $[Cu(phen)(nal)]^+$ to interact with DNA *in vivo* and its capacity to promote the degradation of plasmid and chromosomal DNA, under reductive conditions has been previously reported. However whether this compound utilizes other intracellular targets to promote bacterial killing was a question that deserved to be answered. In this paper, the studies of the chemical nuclease properties encoded by the metal complex $[Cu(phen)(nal)]^+$ were extended by using different types of single chain nucleic acids, i.e, ribosomal and tumor mosaic virus RNAs as well as poly-dA-dT. Our results showed that degradation of the nucleic acids occurred only under reductive conditions. Although MPA and β-mercaptoethanol were the chemical reducers that best assisted the nuclease reaction, other biological compounds such as citric and succinic acid also were shown to act like reducers in that reaction. Although the nuclease activity of $[Cu(phen)(nal)]^+$ was comparable to that exhibited by bis copper phenanthroline $[Cu(phen)_2]^{2+}$our results showed that none of the individual components of $[Cu(phen)(nal)]^+$ was able to promote the degradation of either the RNAs or poly(dA-dT). These results strongly support the hypothesis that the metal complex $[Cu(phen)(nal)]^+$ uses not only DNA but also RNA as targets to promote bacterial killing.

---

✉ Tel: + 52 (473) 73 200 06 (Ext. 8161 & 8173) Fax: +52 (473) 73 200 06 (Ext. 8153).
E. mail: pedrama@quijote.ugto.mx

**Abbreviations**: **DTT** (dithiothreitol); **MPA** (mercaptopropionic acid); **nal** (nalidixate); **TMV RNA** (tobacco mosaic virus RNA); **EDTA** (ethylenediaminetetraacetic acid).

### INTRODUCTION

Intracellular metals strongly influence the activity of quinolones due to dramatic effects on their physicochemical properties /1/. To investigate how the transition metals exert their action on biological systems, the coordinative behavior of quinolone drugs in complexes of the type $[Cu(phen)(antib)]^+$ (where antib is a quinolone or fluoroquinolone) has been explored /2,3/. This approach has allowed altering not only the potency but also the specificity of quinolones /4,5/.

The metal complex $[Cu(phen)(nal)]^+$ (where nal is nalidixate; Fig. 1) was previously used as a model to investigate how this type of compound affects bacterial viability /4, 5, 6/. Our results demonstrated that this compound not only possessed the ability of interacting with DNA *in vivo* but also was shown to encode a potent chemical nuclease which under reductive conditions promoted the degradation of plasmid and chromosomal DNA /6/.

Whether $[Cu(phen)(nal)]^+$ uses only DNA as its intracellular target remained to be investigated, due to the fact that in addition to degrading bacterial genomic DNA, $[Cu(phen)(nal)]^+$ also exhibited the ability to attack ribosomal RNA /6/. The relevance of this observation regarding the bacterial intracellular targets of this compound prompted us to characterize its nuclease activity during the degradation of single stranded nucleic acids.

We report here the characterization of the chemical nuclease activity of $[Cu(phen)(nal)]^+$ using as substrates two types of single chain nucleic acids , i.e, ribosomal and tumor mosaic virus RNAs as well as poly (dA-dT).

The results obtained here support the hypothesis that the metal complex $[Cu(phen)(nal)]^+$ exerts its activity against bacteria not only through the interference with DNA metabolic processes but also by promoting the degradation of other nucleic acids like messenger and ribosomal RNAs.

### MATERIAL AND METHODS

### Synthesis of $[Cu(phen)(nal)]^+$

Synthesis and purification of the metal complex $[Cu(phen)(nal)(H_2O)]NO_3{\cdot}3H_2O$ (Fig. 1) was carried out as previously described /2, 3/. Copper phenantroline $[Cu(phen)_2]^{2+}$ was prepared in solution by mixing equimolar amounts of copper(II) nitrate and phenanthroline previously dissolved in 20 ml of distilled water to a final concentration of 1mM.

### Reagents and Enzymes

All the chemicals used were of analytical grade. The chemical compounds 1,10-phenanthroline, copper nitrate and nalidixic acid were obtained from Merck, J.T. Baker and Sigma Chemical Co., respectively.

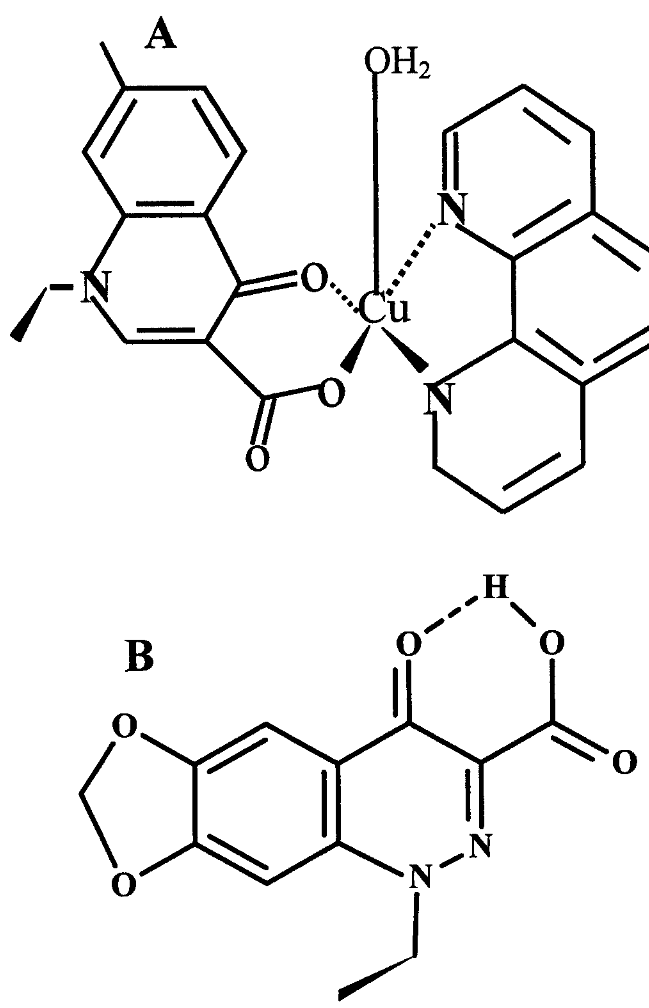

**Fig. 1**: Chemical Structure of nalidixic acid (nal) (**B**) and the cationic complex of nalidixate, phenatroline copper $^{(II)}$ $[Cu(phen)(nal)]^+$ (**A**) in the solid state

### Physical Damage of Nucleic Acids by $[Cu(phen)(nal)]^+$

Experiments to characterize the nuclease activity of $[Cu(phen)(nal)]^+$were carried out with three different type of substrates, i.e, bacterial ribosomal RNA (rRNA), tobacco mosaic virus RNA (TMV RNA) and poly (dA-dT). rRNA was isolated from stationary cultures of the strain *Escherichia coli* DH5α following the protocol of alkaline lysis /7/. The 6350 bases-long tobacco mosaic virus RNA and poly (dA-dT) were purchased from Boehringer Manheim. A typical reaction mixture contained, in a final volume of 25 μl, nucleic acid, 250 ng; $[Cu(phen)(nal)]^+$, different concentrations; and mercaptopropionic acid (MPA), 7 mM. The reaction mixture was incubated for 60 min at 37°C and then diluted 1:6 with loading buffer, which contained 40 mM Tris-acetate buffer, pH 8, 1 mM EDTA, 12% (v/v) glycerol, and bromophenol blue as a tracking dye, and loaded on a 1% agarose gel containing 40 mM tris-acetate buffer, pH 8.0, 1 mM EDTA (TAE), and 0.04 mM ethidium bromide. The gel was run in the same buffer at 90 volts. The nucleic acids on the gel were observed and photographed using an Eagle Eye gel documentation system (Stratagene, USA).

### RESULTS

Previous studies showed that $[Cu(phen)(nal)]^+$ possessed activity of double stranded nuclease [6]. To investigate whether $[Cu(phen)(nal)]^+$ is able to promote the degradation of other type of nucleic acids, the metal complex was first incubated *in vitro* with different concentrations of rRNA isolated from *E. coli*. The results shown in Figure 2 demonstrated that the metal complex $[Cu(phen)(nal)]^+$ at a final concentration of 320 µM, was incapable *per se* of promoting the degradation or inducing any other apparent physical effect on the structure of the rRNA (Fig. 2, Lane 1). However, upon addition of MPA, the metal complex was able to promote the degradation of this type of nucleic acid; such a reaction was concentration dependent and reached a maximum at 80 µM (Fig. 2, Lanes 4-10). The kinetics of degradation of rRNA by $[Cu(phen)(nal)]^+$ was also determined. The results depicted in Figure 3 demonstrate that the ribonuclease reaction was very fast, reaching a maximum after the 5 min of incubation; no further degradation occurred even after 60 min of incubation.

In order to investigate which are the structural requirements of $[Cu(phen)(nal)]^+$ to promote the degradation of rRNA, each of the components of the metal complex at a final concentration of 40 µM was incubated with rRNA in the presence or absence of MPA. As shown in Figure 4, none of the components was capable of degrading the rRNA, but $[Cu(phen)_2]^{2+}$ which caused a level of degradation similar to that exhibited by$[Cu(phen)(nal)]^+$ (Fig. 4, Lanes 4 and 6).

To gain further insight into the chemical nuclease specificity of $[Cu(phen)(nal)]^+$, TMV RNA was used as substrate. The TMV RNA, a 6350-nucleotide single stranded molecule encoding a poly-cistronic messenger RNA was shown to be a substrate for the nuclease activity encoded by $[Cu(phen)(nal)]^+$. As observed for rRNA, the metal complex alone possessed no activity against TMV RNA. However in the presence of MPA, the metal complex promoted the degradation of this type of nucleic acid; as shown in Figure 4 the maximum levels of degradation occurred at a final concentration of 40 µM. Although MPA alone was shown to have a low nuclease activity against this type of RNA (Fig. 5, Lane2), the level of degradation was much lower than

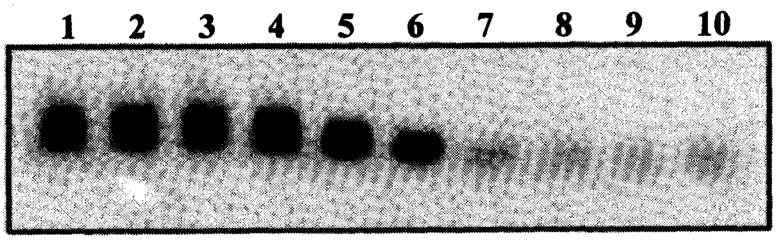

| | | 1 | 2 | 3 | 4 | 5 | 6 | 7 | 8 | 9 | 10 |
|---|---|---|---|---|---|---|---|---|---|---|---|
| RNA | ( 6. 54 mg ) | + | + | + | + | + | + | + | + | + | + |
| $[Cu(phen)(nal)]^+$ | ( mM ) | - | - | **320** | **5** | **10** | **20** | **40** | **80** | **160** | **320** |
| MPA | ( 7 mM ) | - | + | - | + | + | + | + | + | + | + |

**Fig. 2:** Activity of $[Cu(phen)(nal)]^+$ for cleavage of rRNA. rRNA (6.5 µg) was incubated with different concentrations of $[Cu(phen)(nal)]^+$ in the presence (lanes 2, 4-10) or absence (lanes 1 and 3) of MPA (7 mM). Lane 2 The reactions were incubated for 60 min at 37 °C and then analyzed in a 1% agarose gel containing ethidium bromide.

| | | 1 | 2 | 3 | 4 | 5 | 6 | 7 | 8 | 9 | 10 | 11 | 12 | 13 | 14 | 15 | 16 |
|---|---|---|---|---|---|---|---|---|---|---|---|---|---|---|---|---|---|
| RNA | (6.54μg) | + | + | + | + | + | + | + | + | + | + | + | + | + | + | + | + |
| $[Cu(phen)(nal)]^+$ | (40μM) | - | + | - | + | - | + | - | + | - | + | - | + | - | + | - | + |
| MPA | (70mM) | - | - | + | + | - | - | + | + | - | - | + | + | - | - | + | + |
| Time of incubation | (min) | 5 | 5 | 5 | 5 | 10 | 10 | 10 | 10 | 30 | 30 | 30 | 30 | 60 | 60 | 60 | 60 |

**Fig. 3:** Kinetics of rRNA cleavage by $[Cu(phen)(nal)]^+$. Reaction mixtures containing 6.5 μg of rRNA were incubated at 37 °C with (lanes 3, 4, 7, 8, 11, 12, 15, 16) or without (lanes 1, 2, 5, 6, 9, 11, 13, 15) MPA (7mM), in the presence (lanes 2, 4, 6, 8, 10, 12, 14, 16) or in the absence (lanes 1, 3, 5, 7, 9, 11, 13, 15) of $[Cu(phen)(nal)]^+$ (40 μM), for 5 (lanes 1-4), 10 (lanes 5-8), 30 (lanes 9-12) or 60 min (lanes 13-16). The reactions were stopped by addition of loading buffer and analyzed in a 1% agarose gel containing ethidium bromide.

| | | 1 | 2 | 3 | 4 | 5 | 6 | 7 | 8 | 9 | 10 | 11 | 12 | 13 | 14 | 15 | 16 |
|---|---|---|---|---|---|---|---|---|---|---|---|---|---|---|---|---|---|
| RNA | (6.54 μg) | + | + | + | + | + | + | + | + | + | + | + | + | + | + | + | + |
| MPA | (7 mM) | - | + | - | + | - | + | - | + | - | + | - | + | - | + | - | + |
| $[Cu(phen)(nal)]^+$ | (40 μM) | - | - | + | + | - | - | - | - | - | - | - | - | - | - | - | - |
| $[Cu(phen)_2]^{2+}$ | (40 μM) | - | - | - | - | + | + | - | - | - | - | - | - | - | - | - | - |
| Cu-nal | (40 μM) | - | - | - | - | - | - | + | + | - | - | - | - | - | - | - | - |
| phen-nal | (40 μM) | - | - | - | - | - | - | - | - | + | + | - | - | - | - | - | - |
| $Cu(NO_3)_2$ | (40 μM) | - | - | - | - | - | - | - | - | - | - | + | + | - | - | - | - |
| phen | (40 μM) | - | - | - | - | - | - | - | - | - | - | - | - | + | + | - | - |
| nal | (40 μM) | - | - | - | - | - | - | - | - | - | - | - | - | - | - | + | + |

**Fig. 4:** Activity of $[Cu(phen)(nal)]^+$ and each of its components toward rRNA. rRNA (6.54 μg)) was incubated with either $[Cu(phen)(nal)]^+$ (lanes 3,4) ,$Cu(fen)_2$ (lanes 5,6), Cu(nal) (lanes 9,10), copper nitrate (lanes 11,12), phen (lanes 13,14) or nalidixic acid (lanes 15,16) each at a final concentration of 10μM, in the presence (lanes 2, 4 ,6, 8, 10, 12, 14, 16) or in the absence (1, 3, 5,7, 9, 11, 13, 15) of MPA (7 mM). Reactions were incubated for 60 min at 37 °C and then analyzed in a 1% agarose gel containing ethidium bromide.

that exhibited by the metal complex when this was added to the reaction to different concentrations (Figure 5, Lanes 4-11).

The other type of nucleic acid tested here was poly (dA-dT), a single stranded polymer, which has the ability of forming double strands of variable sizes and whose structure alternates the bases deoxy adenine and deoxy thymine. Results depicted in Figure 6 revealed that this type of DNA is also degraded by the metal complex $[Cu(phen)(nal)]^+$, this nuclease reaction was shown to be concentration dependent and only occurred

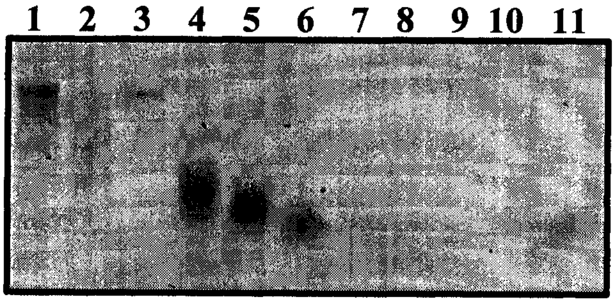

| | | 1 | 2 | 3 | 4 | 5 | 6 | 7 | 8 | 9 | 10 | 11 |
|---|---|---|---|---|---|---|---|---|---|---|---|---|
| TMV RNA | (250 ng) | + | + | + | + | + | + | + | + | + | + | + |
| MPA | (7 mM) | - | + | - | + | + | + | + | + | + | + | + |
| $[Cu(phen)(nal)]^+$ | (μM) | 0 | 0 | 640 | 5 | 10 | 20 | 40 | 80 | 160 | 320 | 640 |

**Fig. 5:** Activity of $[Cu(phen)(nal)]^+$ for cleavage of TMV RNA. TMV RNA (250 ng) was incubated with different concentrations of $[Cu(phen)(nal)]^+$ in the presence (lanes 2, 4-11) or absence (lanes1, 3) of MPA (7 mM). The reactions were incubated for 60 min at 37 °C and then analyzed in a 1% agarose gel containing ethidium bromide.

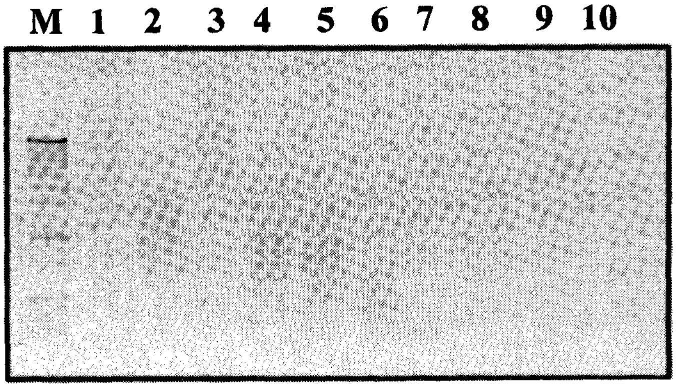

| | | 1 | 2 | 3 | 4 | 5 | 6 | 7 | 8 | 9 | 10 |
|---|---|---|---|---|---|---|---|---|---|---|---|
| Poly dA - dT | (250 ng) | + | + | + | + | + | + | + | + | + | + |
| MPA | (7 mM) | - | + | - | + | + | + | + | + | + | + |
| $[Cu(phen)(nal)]^+$ | (μM) | 0 | 0 | 320 | 5 | 10 | 20 | 40 | 80 | 160 | 320 |

**Fig. 6:** Activity of $[Cu(phen)(nal)]^+$ for cleavage of poly (dA-dT). Poly (dA-dT) (250 ng) was incubated with different concentrations of $[Cu(phen)(nal)]^+$ in the presence (lanes 2, 4-10) or absence (lanes1, 3) of MPA (7 mM). The reactions were incubated for 60 min at 37 °C and then analyzed in a 1% agarose gel containing ethidium bromide.

in the presence of the reducing agent MPA. As observed during degradation of TMV RNA, MPA alone also was shown to possess activity against poly (dA-dT) (Fig. 6, Lane 2); however, the reaction was much stronger in the presence of $[Cu(phen)(nal)]^+$, reaching a maximum level at a concentration of 40 µM (Fig. 5, lanes 4-10).

Using as a substrate plasmid DNA, our previous results demonstrated that $[Cu(phen)(nal)]^+$ encoded a more powerful chemical nuclease than $[Cu(phen)_2]^{2+}$ /6/. Therefore, in this work the potency of both compounds during degradation of rRNA was also compared. We analyzed this reaction as a function of the concentration of the metal complexes. The results shown in Figure 7 demonstrate that no substantial difference exist between both complexes to promote the degradation of rRNA. In both cases the reaction reached a maximum at a final concentration of 80 µM and was strictly dependent on the presence of the thiol compound MPA.

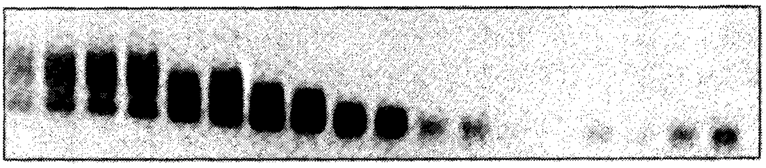

| | | 1 | 2 | 3 | 4 | 5 | 6 | 7 | 8 | 9 | 10 | 11 | 12 | 13 | 14 | 15 | 16 | 17 | 18 |
|---|---|---|---|---|---|---|---|---|---|---|---|---|---|---|---|---|---|---|---|
| RNA | (6.54 µg) | + | + | + | + | + | + | + | + | + | + | + | + | + | + | + | + | + | + |
| [Cu(phen)(nal)] | (mM) | - | - | 320 | - | 5 | - | 10 | - | 20 | - | 40 | - | 80 | - | 160 | - | 320 | - |
| $[Cu(phen)_2]^{2+}$ | (mM) | - | - | - | 320 | - | 5 | - | 10 | - | 20 | - | 40 | - | 80 | - | 160 | - | 320 |
| MPA | (7 mM) | - | + | - | - | + | + | + | + | + | + | + | + | + | + | + | + | + | + |

**Fig. 7:** Comparison of nuclease activity of the metal complexes $[Cu(phen)(nal)]^+$ and $[Cu(phen)_2]^{2+}$ for cleavage of rRNA. rRNA (6.5 µg) was incubated with different final concentrations of either $[Cu(phen)(nal)]^+$ (5 µM, lane 5; 10 µM, lane 7 ; 20 µM, lane 9; 40 µM, lanes 11; 80 µM, lane 13; 160 µM, lane 15; 320 µM, lane 3 and 17) or $[Cu(phen)2]^{2+}$ (5µM, lane 6; 10 µM, lane 8; 20 µM, lane 10; 40 µM, lane 12; 80 µM, lane 14; 160 µM, lane 16; 320 µM, lanes 18 and 4), in the presence (lanes 2, 5, 6, 7, 8, 9, 10, 11, 12, 13, 14, 15, 16, 17, 18) or absence (lanes 1, 3, 4) of MPA (7 mM). The reactions were carried out for 60 min at 37 °C and then analyzed in a 1% agarose gel containing ethidium bromide.

As mentioned above, chemical nucleases such as $[Cu(phen)_2]^{2+}$ induce the degradation of double-stranded DNA in an oxygen-dependent reaction, but only in the presence of a reducing agent such as mercaptopropionic acid (MPA) /8, 9/. The nuclease activity of $[Cu(phen)(nal)]^+$ against both double stranded DNA /6/ and single stranded nucleic acids (this work) has also been shown to be dependent on the presence of MPA. To investigate whether other reducing agents, in addition to MPA, could assist the RNAse activity of $[Cu(phen)(nal)]^+$, the following compounds were tested: succinic acid, tartaric acid, citric, thiolactic acid, as well as β-mercaptoethanol and dithiothreitol (DTT). Results seen in Figure 8 showed that when these compounds were included into the mixture reaction at the same final concentration, (i.e., 7 mM), MPA and β-

mercaptoethanol were the reagents that best assisted the nuclease reaction. However, although to a lesser extent, other biological compounds such as succinate and citrate were also able to assist the ribonuclease reaction of $[Cu(phen)(nal)]^+$.

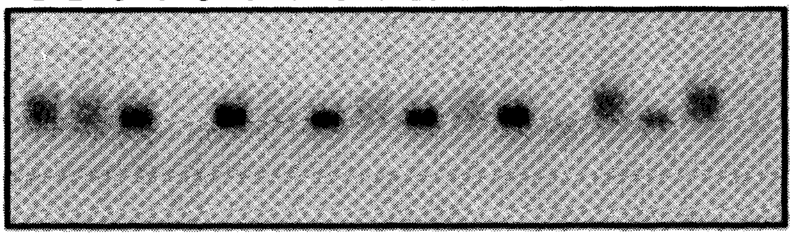

| | | 1 | 2 | 3 | 4 | 5 | 6 | 7 | 8 | 9 | 10 | 11 | 12 | 13 | 14 | 15 | 16 |
|---|---|---|---|---|---|---|---|---|---|---|---|---|---|---|---|---|---|
| RNA | (500 ng) | + | + | + | + | + | + | + | + | + | + | + | + | + | + | + | + |
| $[Cu(phen)(nal)]^+$ | (80 µM) | - | + | - | + | - | + | - | + | - | + | - | + | - | + | - | + |
| MPA | (7 mM) | - | - | + | + | - | - | - | - | - | - | - | - | - | - | - | - |
| Succinic Acid | (7 mM) | - | - | - | - | + | + | - | - | - | - | - | - | - | - | - | - |
| Tartaric Acid | (7 mM) | - | - | - | - | - | - | + | + | - | - | - | - | - | - | - | - |
| Citric Acid | (7 mM) | - | - | - | - | - | - | - | - | + | + | - | - | - | - | - | - |
| Thiolactic Acid | (7 mM) | - | - | - | - | - | - | - | - | - | - | + | + | - | - | - | - |
| DTT | (7 mM) | - | - | - | - | - | - | - | - | - | - | - | - | + | + | - | - |
| β-mercaptoethanol | (7 mM) | - | - | - | - | - | - | - | - | - | - | - | - | - | - | + | + |

**Fig. 8:** Activity of $[Cu(phen)(nal)]^+$ for cleavage of rRNA with different reducing agents. rRNA (500 ng) was incubated with (Lanes 2, 4, 6, 8, 10, 12, 14, 16) or with out (Lanes 1, 3, 5, 7, 11, 13, 15) 80 µM $[Cu(phen)(nal)]^+$ and different reducing agents at a final concentration of 7 mM. The reactions were incubated for 60 min at 37 °C and then analyzed in a 1% agarose gel containing ethidium bromide.

### DISCUSSION

The ability of $[Cu(phen)(nal)]^+$ to interact *in vivo* with bacterial DNA and to promote its degradation *in vitro* has been widely substantiated by our previous studies /6/. In addition to degrading bacterial genomic DNA, $[Cu(phen)(nal)]^+$ also exhibited the ability to attack ribosomal RNA /6/. The implication of this result regarding the bacterial intracellular targets of this compound prompted us to characterize its nuclease activity during the degradation of single stranded nucleic acids.

As previously reported during the studies of the nuclease activity of $[Cu(phen)(nal)]^+$ against plasmid and chromosomal DNA /6/, the metal complex alone was incapable of promoting the degradation or inducing any other apparent physical effect on the structure of either of the single-stranded nucleic acids tested here. This result demonstrates that the compound does not possess nuclease activity *per se*; however, upon addition of the reducing agent MPA, a strong nuclease activity against the three substrates was unmasked. This behavior has been observed not only for $[Cu(phen)(nal)]^+$ but also for $[Cu(phen)_2]^{2+}$ /10/ and other metal complexes whose nuclease activity is triggered by a reducing agent in the presence of molecular oxygen or hydrogen peroxide /10, 11, 12/.

Experiments with different reducing agents showed that in addition to MPA other strong reducing agents, such as beta-mercaptoethanol and thiolactic acid, were also able to support fully the RNAse reaction promoted by $[Cu(phen)(nal)]^{+}$ (Fig. 8). Although to a lesser extent, succinic and citric acid also promoted the RNAse reaction of the metal complex. However, the low power exerted by these biological compounds could have important implications concerning the possible intracellular effects of this compound during its interference with bacterial viability.

Degradation of the three type of nucleic acids tested here was not only dependent on the concentration used, but also on the whole structure of the complex. These results are relevant due to the fact that experimental evidence from physiological studies has demonstrated that the whole compound is also required to induce the maximum levels of the SOS response in *B. subtilis* /6/ and to interfere maximally with the growth of some bacterial species /4, 5/.

$[Cu(phen)(nal)]^{+}$ was shown to possess a stronger nuclease activity than $[Cu(phen)_2]^{2+}$ during degradation of double-stranded DNA /6/. In the present work, we observed no difference between the potency of both metal complexes to degrade either rRNA (Fig. 7), or TMV RNA and poly (dA-dT) (Results not shown). Although at present we have no explanation for these results, it is possible that the different potencies exhibited by $[Cu(phen)(nal)]^{+}$ to degrade double or single stranded nucleic acids could be influenced by the secondary or tertiary structure (or both) adopted by these nucleic acids.

Previous reports demonstrated that $[Cu(phen)_2]^{2+}$ is also capable of cleaving the phosphodiester backbone of RNA /9, 13/. Interestingly, in the RNA cleavage reaction it was observed that single-stranded regions were much more susceptible to scission than double-stranded stem structures /11/. Other chemical nucleases with RNAse activity have been reported, for instance, methyl propidium-EDTA /14/, ferrous-EDTA /15/, and light-activated uranyl acetate /18/. An interesting aspect regarding the mode of action of these last two complexes is that they seem to cut RNA in a primary and secondary structure-independent manner. $[Cu(phen)(nal)]^{+}$ behaves like these compounds as equally promoting the degradation of mRNA and rRNA. Therefore this compound could have a potential application as a footprinting reagent to study the interactions between proteins and DNA as well as with RNA.

The results shown here demonstrate that under reducing conditions, the metal complex $[Cu(phen)(nal)]^{+}$ is able to induce the degradation of two forms of RNA, i.e., ribosomal and messenger RNA. Although the mechanism of cleavage of RNA by $[Cu(phen)(nal)]^{+}$ is a matter that deserves to be further investigated, the implications of these results are very important in terms of the possible bacterial intracellular targets used by this compound to promote bacterial killing. Degradation of rRNA could have a dramatic effect on protein synthesis; a similar effect, with no less dramatic consequences, could be the degradation of messenger RNAs. On the other hand the nuclease activity exerted by $[Cu(phen)(nal)]^{+}$ against poly (dA-dT) could be physiologically relevant, for instance during the establishment of the complex RNA polymerase-open promoter where zones of single stranded DNA are exposed and potentially could be substrates for the attack of the metal complex $[Cu(phen)(nal)]^{+}$. This prediction is not unprecedented, as Spessky and Sigman /17/ reported that $[Cu(phen)_2]^{2+}$ shows a preference to attack the template strand during the establishment of the open promoter lac UV-5 complex with *E. coli* RNA polymerase *lac*UV-5 /17/. Foot printing reactions revealed that $[Cu(phen)_2]^{2+}$ preferentially attacks the positions –6 to –4 upstream of the transcription start site; such sites were shown to be within a single stranded region formed at the active site of RNA polymerase.

Thus, taking into account the results obtained here together with previous studies which demonstrated that $[Cu(phen)(nal)]^+$ interacts with DNA, putatively affecting essential DNA functions such as replication and transcription /6/, we conclude that other nucleic acids such as rRNA and mRNA could be potential intracellular targets during the bacterial killing promoted by $[Cu(phen)(nal)]^+$.

### ACKNOWLEDGEMENTS

This work was supported by grant 31767-N from the Consejo Nacional de Ciencia y Tecnología (CONACYT) of México to M. Pedraza-Reyes. N. Ramírez-Ramírez was supported by a Master of Sciences fellowship from CONACYT.

### REFERENCES

1. G. Mendoza-Díaz, R. Pérez-Alonso and R. Moreno-Esparza. *J Inorg Biochem*, **64**, 207-214 (1996).
2. G. Mendoza-Díaz, L.M.R. Martínez-Aguilera, R. Pérez-Alonso, X. Solans and R. Moreno-Esparza. *Inorg Chim Acta*, **138**, 41-47 (1987).
3. G. Mendoza-Díaz, L.M.R. Martínez-Aguilera, R. Moreno-Esparza, K.H. Pannell and F. Cervantes Lee. *J Inorg Biochem*, **50**, 65-78 (1993).
4. S. Arias-Negrete, M. Nieto-Gómez, F. Anaya-Velázquez, R.M. García-Nieto and G. Mendoza Díaz. *Rev Soc Quim*, **37**, 3-7 (1993).
5. G. Mendoza-Díaz, S. Arias-Negrete, R.M. García-Nieto, L. Ruíz-Ramírez, F. Anaya Velásquez, I. Gracia-Mora and R. Moreno-Esparza. In: Cervantes-Vega C, Saavedra MA, Farias RR (eds). *La importancia biológica de los iones orgánicos*. Universidad Michoacana, Secretaria de difusión cultural/Editorial Universitaria, 1993.
6. N. Ramírez-Ramírez, G. Mendoza-Díaz, F. Gutiérrez-Corona and M. Pedraza-Reyes. *J Biol Inorg Chem*, **3**, 188-194 (1998).
7. J. Sambrook, E.F. Fritsch and T. Maniatis. *Molecular Cloning: A Laboratory Manual*, 2nd edn. Cold Spring Harbor Laboratory, Cold Spring Harbor, N.Y., 1989.
8. D.S. Sigman, D.R. Graham, V. D'Aurora and A.M. Stern. *J Biol Chem*, **254**, 12269-12272 (1979).
9. D.S. Sigman, A. Mazumder and D.M. Perrin. *Chem Rev*, **93**, 2295-2316 (1993).
10. M. Zhao and M.J. Clarke. *J Biol Inorg Chem*, **4**, 325-340 (1999).
11. J.G. Muller, X. Chen, A.C. Dadiz, S.E. Rokita and C.J. Burrows. *Appl Chem*, **65**, 545-550 (1993).
12. M. Pitié and B. Meunier. *J Biol Inorg Chem*, **1**, 239-246 (1996).
13. G.J. Murakawa, C.-H.B. Chen, M.D. Kuwabara, D. Nierlich and D.S. Sigman. *Nucleic Acids Res*, **17**, 5361-5369 (1989).
14. J. Kean, S.A. White and D. Drapper. *Biochemistry*, **24**, 5062-5070 (1985).
15. X. Wang and R.A. Padget. *Proc Natl Acad Sci USA*, **86**, 7795-7799 (1989).
16. R. Gaynor, E. Soultanakis, M. Kuwabara, J. García and D.S. Sigman. *Proc. Natl. Acad. Sci. USA*, **86**, 4858-4863 (1989).
17. A. Spessky and D.S. Sigman. *Biochemistry*, **24**, 8050-8056 (1985).

